# Oral treatment with *Rosa multiflora* fructus extract modulates mast cells in canine atopic dermatitis

**DOI:** 10.3389/fvets.2025.1531313

**Published:** 2025-04-09

**Authors:** Ha-Young Shin, Sang Hun Shin, Hee Soon Shin, Hyun-Jin Tae, Hyun-Jin Kim, Jeong Ho Hwang

**Affiliations:** ^1^Center for Large Animals Convergence Research, Korea Institute of Toxicology, Jeongeup, Republic of Korea; ^2^College of Veterinary Medicine, Jeonbuk National University, Iksan, Republic of Korea; ^3^College of Veterinary Medicine and Institute of Animal Transplantation, Jeonbuk National University, Republic of Korea; ^4^Research Division of Food Functionality, Korea Food Research Institute, Wanju, Republic of Korea; ^5^Department of Food Biotechnology, University of Science and Technology, Daejeon, Republic of Korea; ^6^Department of Food Science and Technology, Gyeongsang National University, Jinju, Republic of Korea; ^7^EZmass Co. Ltd., Jinju, Republic of Korea

**Keywords:** canine atopic dermatitis, *Rosa multiflora* fructus extract, Th2-related response, mast cell regulation, beagle

## Abstract

**Introduction:**

Canine atopic dermatitis is a hereditary, often pruritic, and predominantly T-cell-driven inflammatory skin disease involving an interplay between skin barrier abnormalities and allergen sensitization. However, progress in developing therapeutics for companion animals remains slow, with few drugs advancing to Phase II clinical trials to investigate the underlying mechanisms in target animals. While *Rosa multiflora* fruit extract (RMFE) has been strongly implicated in the improvement of various inflammatory diseases, its effects on canine atopic dermatitis (cAD) and the putative underlying mechanisms remain unclear. In this study, we aimed to evaluate the efficacy of RMFE in the treatment of cAD and explore its underlying mechanisms.

**Methods:**

In this study, RMFE was administered orally (repeatedly for 2 weeks) to ovalbumin (OVA)-induced atopic dermatitis-induced beagles. The effects of RMFE on cAD were assessed through clinical symptom observation and scoring using the canine atopic dermatitis extent and severity index. Additionally, histopathological analysis was performed (hematoxylin and eosin, Masson’s trichrome, and toluidine blue). Cluster of differentiation 4-positive immunostaining was also performed, along with cytokine level and messenger ribonucleic acid level analyses of T-helper 2 (Th2) immune and inflammatory response markers in the modeled skin.

**Results:**

RMFE improved the clinical manifestations of cAD, leading to histopathological modulation of inflammation and immune cells. It also altered Th2 effector cytokine levels. Furthermore, RMFE reduced allergic responses in the AD model dogs by reducing mast cell numbers, inhibiting their activation to release inflammatory mediators, and reducing immunoglobulin E (IgE) production.

**Discussion:**

Our results suggest that RMFE can modulate mast cell activation and Th2-dominant immune responses in cAD, helping to reduce AD-induced inflammatory responses.

## 1 Introduction

Canine atopic dermatitis (cAD) is an inflammatory skin disease characterized by recurrent inflammation and itching (1, 2). The pathogenesis of atopic dermatitis is complex, involving genetic, environmental, and various immunological factors (3–6). In cAD, allergens entering the respiratory tract or skin mucosa are captured and sensitized by antigen-presenting cells after crossing the skin barrier. This initial sensitization activates naïve T cells and induces T-helper (Th)1 and Th2 differentiation in a cluster of differentiation 4-positive (CD4+) T cells. This is important for the progression of allergy (7). Th2-dominant differentiation of CD4+ T helper cells is induced by allergens, and activation of Th2 cells results in the production of effector cytokines, such as (IL)-4, IL-5, and IL-13. These cytokines promote isotype switching and immunoglobulin (Ig)E production when Th2 effector cells interact with allergen-specific B cells (8–11). Additionally, they contribute to the development, activation, and tissue infiltration of eosinophils, further amplifying the allergic response. IgE binds to mast cells via the high-affinity FcεRI receptor, and upon re-exposure to the allergen, it triggers degranulation and the release of chemical inflammatory mediators (e.g., histamine), triggering the hypersensitivity reaction and the clinical manifestations of atopic dermatitis (12). Thus, the Th1/Th2 imbalance, characterized by elevated levels of Th2 cytokines and increased eosinophilic inflammation, is an important marker in the progression of allergic atopic dermatitis (13, 14).

*Rosa multiflora* fruit extract (RMFE) is commonly used as a food and herbal remedy due to its low toxicity (15). It has been traditionally used in herbal medicine for various human conditions, such as inflammatory diseases (skin diseases (16), edema (17), and arthritis (18)) and the common cold. Previous studies have reported the anti-inflammatory properties of RMFE in rodent models of atopic dermatitis (19–21). They also reported that RMFE induces antigen-specific T-cell activation, inhibits proliferation, contributes to the reduction of Th2 cytokine production, modulates mast cell function, and inhibits pro-inflammatory cytokine production. Given the efficacy of RMFE against various inflammatory diseases, it was expected to have a therapeutic effect on cAD. In this study, we aimed to investigate the efficacy of RMFE in inhibiting the progression of canine atopic-like skin inflammation. In addition to measuring hematological parameters, inflammatory cytokine levels, total blood IgE, and OVA-specific IgE levels, we evaluated gross observations of the skin (canine atopic dermatitis extent and severity index) and histological changes to assess RMFE’s modulation of Th2-related immune responses in atopic dermatitis and to explore its anti-atopic mechanisms.

## 2 Materials and methods

### 2.1 Animals

Eight beagles (*Canis lupus familiaris*), consisting of four males and four females, aged 1–2 years, with a mean weight of 7.95 kg (range: 7.2–8.7), were used in this experiment. The dogs were obtained from Raon Bio (Yongin, Republic of Korea). Before the experiment, a 7-day acclimation period was followed by veterinary quarantine. Only healthy animals that did not develop dermatitis or allergic diseases during quarantine were included in the study. Each animal was housed in cages (130 × 312 × 125 cm^3^) during the dosing period. The experiment maintained room temperature and humidity at 20–26°C and 30–70%, respectively. Fluorescent light intensity was maintained at 300 lux and above, with an air exchange rate of 10–20 times/h and a 12-h light cycle. All animals received daily treats (jerky) and toys (DNA flexer) and were single-housed for the study to prevent variations due to skin injury on the exposed dorsal area. To compensate for this and to minimize the stress on the beagles, who are social animals, they were given additional interaction time with trained technicians. Bathing was avoided during the study to prevent altering skin condition parameters. All animal experiments complied with the guidelines of the Institutional Animal Care and Use Committee of the Korea Institute of Toxicology (KIT) (IACUC-24-01-0107-0068).

### 2.2 Study design

**Figure 1:**
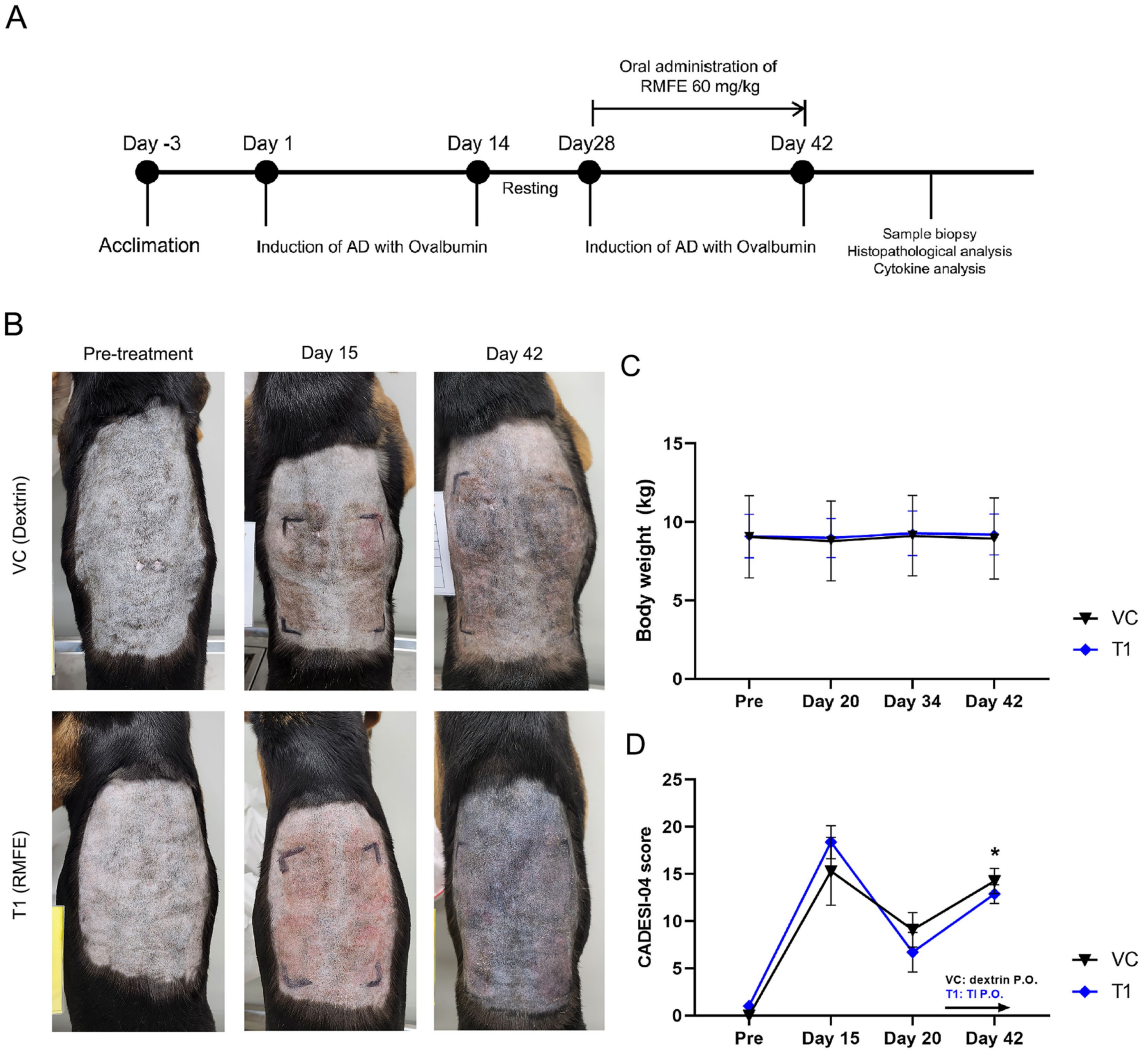
RMFE improves clinical signs in canine AD skin lesions. **(A)** Experimental design of canine AD model and RMFE treatment. **(B)** All animals were evaluated by visual observation and CADESI-04 scoring during the study. Gross observation images and scores were obtained before ovalbumin administration (‘pre-treatment’), after primary sensitization (‘Day 15’), and after secondary ovalbumin stimulation and RMFE treatment (‘Day 42’). **(C)** Body weight was measured to determine the stress caused by oral administration during the test period. **(D)** Summary of CADESI-04 scoring results, *N* = 4, *p*-value: * <0.05.

A model of ovalbumin-induced cAD was established as previously described (22). Briefly, beagles were epicutaneously exposed to 1 mg/kg of ovalbumin at the dorsal four sites once daily for 2 weeks, followed by a second round of 1 mg/kg ovalbumin administered epicutaneously once daily after an incubation period of approximately 2 weeks. Simultaneously with the second simulation of ovalbumin, the treatment group (T1) was treated with RMFE extract (60 mg/kg) in gelatin capsules, administered orally once daily for 2 weeks. The vehicle control (VC) group received 50% dextrin in gelatin capsules, also administered orally. Each group included two male and two female dogs. The control group included dogs with no ovalbumin exposure on their dorsal regions (Figure 1A).

### 2.3 Canine atopic dermatitis extent and severity index (CADESI-04)

The Canine Atopic Dermatitis Degree and Severity Index (CADESI-04) is a clinical scoring system that assesses the severity of skin lesions in dogs with atopic dermatitis and is an established method for evaluating improvement in skin lesions (23). Symptom severity was assessed using a simplified version of the CADESI-04, which assesses erythema, lichenification, and alopecia/keratinization in dermatological studies. Higher scores indicate atopic dermatitis caused by ovalbumin. The version of CADESI-04 administered in this study focused on the dorsal region, the modeling site for cAD, by dividing the back into four sections, scoring each section separately, and summing the scores (23). The CADESI-04 assessments were conducted at four points: before the test, after the primary desensitization, before the secondary stimulus, and after the test substance and secondary stimulus. Scoring was as follows: 0 (no erythema, keratinization, or hair loss); 1 (barely noticeable erythema, keratinization, or hair loss); 2 (clearly visible erythema, keratinization, or hair loss); 3 (moderate to severe erythema, keratinization, or hair loss); and 4 (severe erythema, keratinization, or hair loss, deep tissue damage, and severe edema beyond the exposed area). Three veterinarians and an experienced laboratory technician conducted the CADESI-04 evaluations.

### 2.4 Sample collection and analysis

On day 42, after cAD modeling and RMEF treatment, whole blood was collected in anticoagulant ethylenediamine tetraacetic acid (EDTA) and serum-separating tubes (SST) for complete blood count and blood chemistry, respectively. A complete blood count analysis (BC-2800 Vet; Mindray, Shenzhen, China) was performed using whole blood samples. SST samples were centrifuged for 10 min at 3,000 rpm, and the supernatant was collected as a serum for analysis (DRI-CHEM NX700; Fujifilm, Tokyo, Japan).

### 2.5 Histopathology

On day 42, all animals were anesthetized to obtain skin biopsies using a 6-mm biopsy punch after clinical evaluation and monitoring of skin conditions. Four samples were collected from each dorsal region. For histopathological examination, 50% of the skin samples were fixed in 10% neutral-buffered formalin, and the other 50% were instantly frozen in liquid nitrogen for cytokine analysis. Skin tissues were preserved in 10% neutral buffered formalin overnight, embedded in paraffin, and sectioned at 5 μm intervals. The sections were then deparaffinized and stained with hematoxylin and eosin (H&E) to examine skin structural abnormalities. Masson’s trichrome (MT) staining was performed following the manufacturer’s instructions (Cat. No. IFU-2; ScyTek, Logan, UT, USA). Toluidine blue staining was conducted to assess degranulation of mast cells.

### 2.6 Immunohistochemistry

CD4+ lymphocyte expression was detected by immunohistochemistry using a rabbit monoclonal anti-CD4+ antibody (cat. no. ab133616; 1:100 dilution; Abcam). Deparaffinization of paraffin-fixed skin was performed, while hydration was performed by incubation with Histoclear and graded ethanol concentrations. Antigen retrieval was performed for 30 min. To inhibit endogenous peroxidase activity, skin sections were treated with 3% hydrogen peroxide in phosphate-buffered saline (PBS) for 5 min. The sections were then blocked with 2.5% normal horse serum (cat. no. S-2012; Vector Laboratories) in PBS for 1 h at room temperature. The blocked skin sections were incubated with monoclonal CD4+ antibodies overnight at 4°C and then incubated with peroxidase-conjugated secondary antibodies (catalog no. BP-1400-50; 1:50; Vector Laboratories) for 1 h at room temperature. The sections were treated with diaminobenzidine tetrachloride (cat. no. SK-4105; Vector Laboratories), thoroughly dried, mounted with water-soluble mounting solution, and coverslipped. All sections were photographed using a light microscope (Leica, Wetzlar, Germany), and the images were analyzed with a DHYANA microscope camera.

### 2.7 Quantitative real-time polymerase chain reaction (qRT-PCR)

To quantify ribonucleic acid (RNA) in the samples, RNA was extracted from skin biopsy tissues using the RNeasy Mini Kit (Qiagen, Hilden, Germany) and reverse transcribed using the QuantiNova Reverse Transcription Kit (Qiagen, Hilden, Germany). For quantitative real-time polymerase chain reaction (PCR), 20 μL reaction mixtures were prepared, containing 2 × Power SYBR Green PCR Master Mix (Applied Biosystems, Waltham, MA, USA). The reactions were conducted on a QuantStudio 5 Real-Time PCR System (Applied Biosystems). Glyceraldehyde-3-phosphate dehydrogenase (GAPDH) served as an endogenous control for normalization. Fold induction was quantified using the 2^−ΔΔCT^ method. The sequences for IL-4, IL-5, IL-13, IL-31, Filaggrin, TNF-*α*, IL-1β, CCL17, and GAPDH are listed in Table 1.

**Table 1:** Canine primers used for quantitative real-time PCR.

Gene symbol	Primer sequences (from 5′ to 3′)	Tm (°C)	Gene Bank ID
*IL-4*	F: CATCCTCACAGCGAGAAACG	58	AF054833
	R: CCTTATCGCTTGTGTTCTTTGGA		
*IL-5*	F: GCCTATGTTTCTGCCTTTGC	60	NM_001006950.1
	R: GGTTCCCATCGCCTATCA		
*IL-13*	F: GAATCAGGCATCCCTCTGCA	60	AF244915.1
	R: ATGCCGGCGGTCAGGT		
*IL-31*	F: TGTGCCTGCAGATACTTTTGA	60	AB455159.1
	R: GGTTCGACCAGATAGCCTTG		
*Filaggrin*	F: GATGACCCAGACACTGCTGA	60	XM_038423227.1
	R: TGGTTTTGCTCTGATGCTTG		
*TNF-α*	F: GAGCCGACGTGCCAATG	60	NM_001003244.4
	R: CAACCCATCTGACGGCACTA		
*IL-1β*	F: CACATGAGCTTTGTGCACGG	61	XM_038690464.1
	R: GTAGGGTGGGCTTTCCATCC		
*CCL17*	F: GGCTGACAAGGTGGTACAAGACTTC	60	NM_001003051.1
	R: CAGATGGACTTGCCTTGGACAG		
*GAPDH*	F: TGTCCCCACCCCCAATGTATC	60	NM_001003142
	R: CTCCGATGCCTGCTTCACTACCTT		

### 2.8 Enzyme-linked immunosorbent assay (ELISA)

To analyze cytokines in blood samples, whole blood was collected in anticoagulant EDTA tubes, centrifuged for 10 min at 3,000 rpm, and the supernatant was collected as plasma. All steps were conducted at 2–8°C. Canine IgE, ovalbumin-specific IgE, IL-4, and IL-31 levels were measured using enzyme-linked immunosorbent assay (ELISA) kits (Cat. No. ab157700 for IgE; Abcam, Cambridge, UK; Cat. No. E08O0012–96 for Ovalbumin-specific IgE; Cat. No. DY754 for IL-4), following the manufacturer’s protocols.

### 2.9 Statistical analysis

Statistical analysis was performed using Prism 8 software (GraphPad Software, San Diego, CA, USA). Data are presented as mean ± standard deviation (SD) in all graphs. All experiments were conducted in triplicate. Comparisons between groups were performed using one-way and two-way analysis of variance, and Tukey’s multiple comparison test was used for post-validation. Statistical significance was set at a *p*-value of less than 0.05.

## 3 Results

### 3.1 RMFE improves dermatological clinical signs in canine AD skin lesions

The body weight of the cAD models was measured weekly to assess the stress associated with oral administration. Consequently, no weight loss was observed in either the excipient or RMFE groups following oral administration (Figure 1C). Furthermore, there was no change in food intake, as the animals were provided with a restricted diet typical for stressed animals (data not shown). To ascertain the impact of RMFE on cAD, a visual assessment of the modeled skin lesions was conducted using a simplified clinical evaluation based on the CADESI-04 scoring system (Figures 1B,D). This system evaluated the severity of erythema, lichenification, pigmentation, alopecia, and excoriation in the four dorsal and ventral regions of the body susceptible to atopic dermatitis. The scoring system utilized a four-point scale, with normal conditions assigned a value of 0, mild conditions a value of 1, moderate conditions a value of 2, and severe conditions a value of 3. These values were summed to obtain a total score for each condition. In the initial administration of ovalbumin to induce atopic and allergic dermatitis, erythema, lichenification/pigmentation, alopecia, and excoriation of the dorsal region were observed in all animals. The CADESI-04 scores for the atopic and allergic dermatitis model were as follows: pre-VC: 0.0 ± 0.0, T1: 1.0 ± 0.0; post-VC (Day 8): 15.3 ± 3.6, T1: 18.4. ±1.7; resting period (Day 20): VC: 9.1 ± 1.8, T1: 6.7 ± 2.1; and immediately after the conclusion of the test article administration (Day 42): VC: 14.3 ± 1.3, T1: 12.9 ± 1.0.

### 3.2 RMFE attenuates mast cell and CD4^+^ T cell infiltration and inflammation in canine AD skin lesions

**Figure 2:**
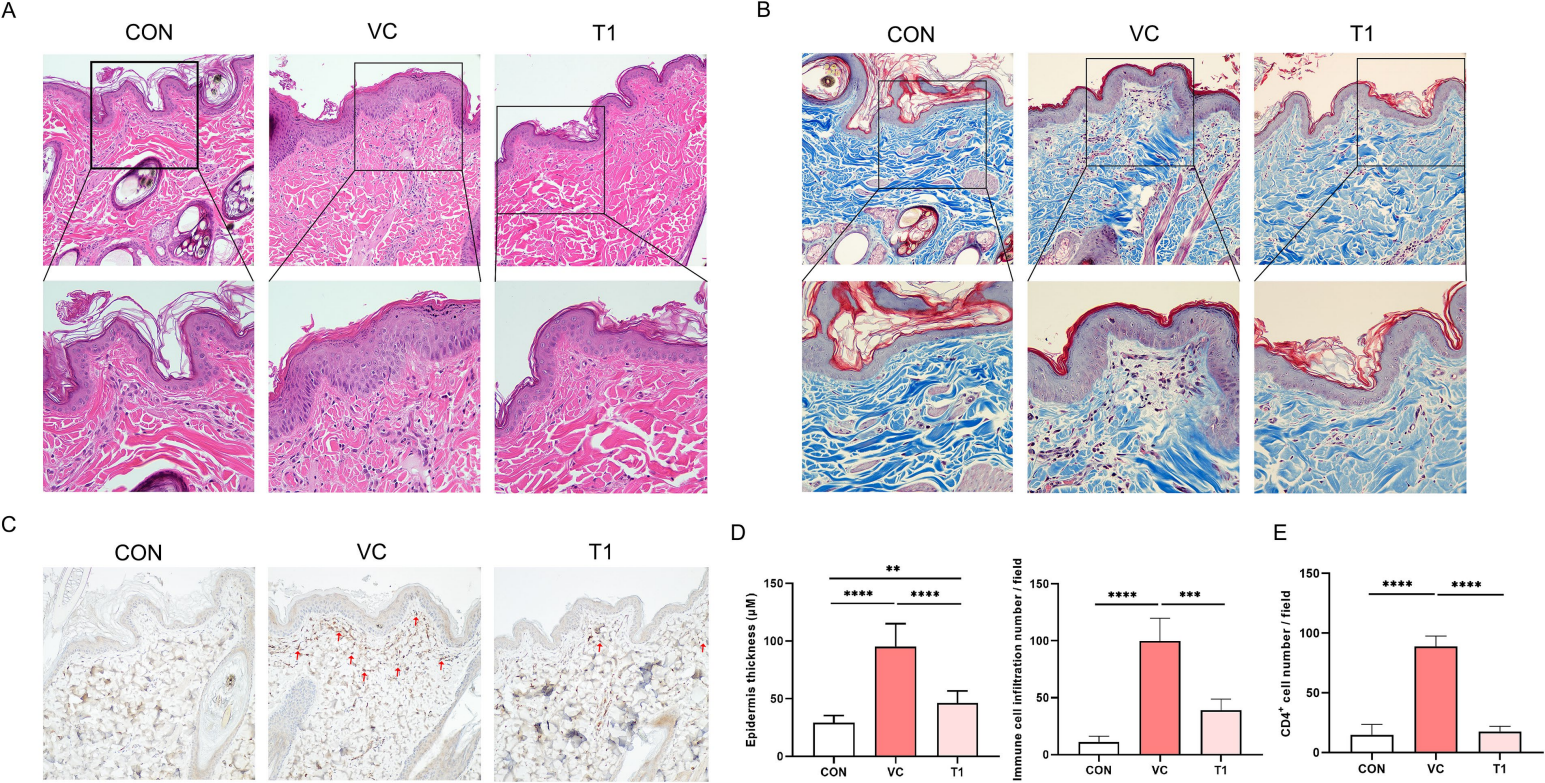
RMFE improves histopathological and immunological signs in canine AD skin lesions. To determine the effect of RMFE on skin histopathological and immunohistological changes, skin biopsies were taken from canine AD modeling sites (VC and T1) and non-modeling sites (CON). **(A)** Skin H&E and **(B)** Masson’s trichrome analysis showed increased epidermal thickness and increased peri-epidermal immune cells in AD modeling sites compared to non-modeling sites. This was alleviated in the RMFE-treated T1 group. Scale bar, 100 μm. **(C)** Immunohistochemical staining of CD4+; **(D)** comparison of skin epidermal thickness and number of immune cell infiltrates; **(E)** infiltration of CD4+ in the peri-epidermis of the skin (red arrows). *p*-values: ** < 0.01, *** < 0.005, **** < 0.001; Bar graph: Mean ± SD.

To determine the histopathological effects of RMFE on cAD skin, dorsal and ventral tissues were biopsied after all treatments. The obtained tissues were subjected to H&E and MT staining to evaluate changes in epidermal morphology, thickness, and immune cell infiltration (Figures 2A–E). In the VC group, epidermal thickness of atopic areas induced by ovalbumin application was significantly increased—more than 3-fold—compared to non-induced atopic areas in the VC group. Specifically, epidermis thickness of the control (CON) group (non-modeled site) exhibited: 29.2 ± 6.0 μm, while epidermis thickness of the VC (modeled site) exhibited 92.1 ± 20.0 μm.

In addition, the epidermal thickness was significantly reduced in the T1 group compared to the VC (modeled site) group, with a mean thickness of 92.1 ± 20.0 μm and 46.2 ± 10.4 μm in the VC and T1 groups, respectively. Given that the average epidermal thickness of CON (non-ovalbumin treated site) was 29.1 μm, oral administration of RMFE was associated with suppression of the atopic-induced increase in epidermal thickness.

Furthermore, immune cells were quantified in four randomly selected fields at 40x magnification to ascertain their potential role in inhibiting immune cell (neutrophil and monocyte) infiltration. In the VC group, the number of infiltrating immune cells at the modeling site was significantly increased—approximately 9-fold—compared to the non-atopic site CON. The mean value for the VC group (modeling site) was 99.7 ± 18.6, compared to 11.0 ± 4.5 for the CON group. Immune cell infiltration was significantly diminished in the RMFE treatment group, with a mean value of 99.7 ± 18.6 for VC and 39 ± 9.1 for T1 (Figures 2A,B,D). In addition, CD4+ cell levels were quantified (Figures 2C,E). Ovalbumin application resulted in increased CD4+ T cell expression in the epidermis and dermis of the VC group compared to the ovalbumin-applied control area (CON). Conversely, RMFE decreased the number of CD4+ T cells.

### 3.3 RMFE regulates allergic inflammation by inhibiting IgE expression and mast cell activation in canine AD

**Figure 3:**
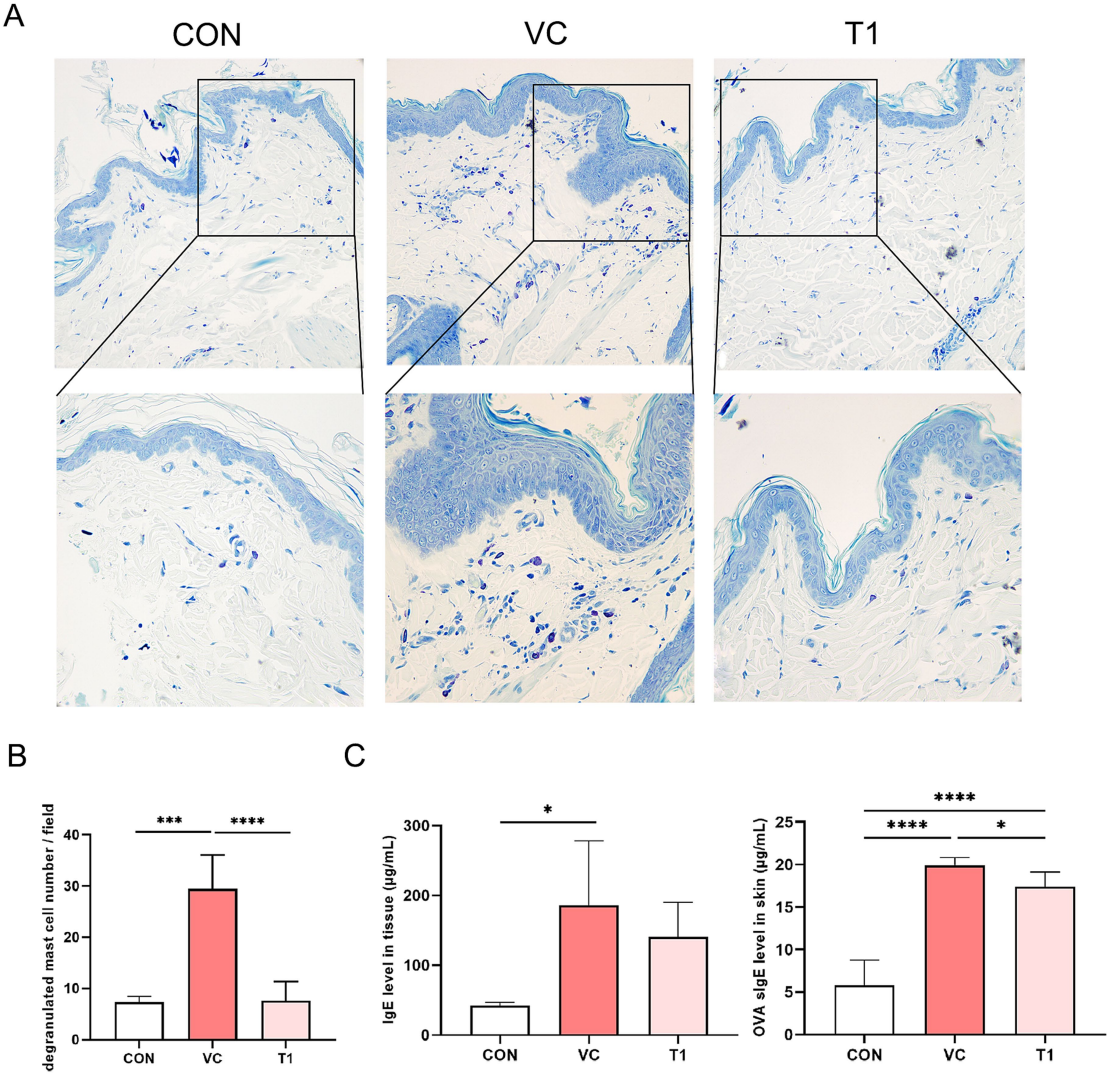
RMFE modulates allergic inflammation by inhibiting IgE expression and mast cell activation in canine AD. Comparative analysis of mast cell degranulation expression changes in canine AD after treatment in all groups. **(A)** Representative images of toluidine blue staining results after RMFE treatment in canine AD modeling sites (VC and T1) and non-modeling sites (CON). Purple staining indicates mast cells. Upper magnification 200x, lower magnification 400x. **(B)** Stained mast cells were counted and graphed in four fields per animal, *N* = 4/group. **(C)** Analysis of total IgE and OVA-specific IgE expression after RMFE treatment in canine AD modeling sites (VC T1) and non-modeling sites (CON). *p*-values: * < 0.05, *** < 0.005, **** < 0.001; Bar graph: Mean ± SD.

To better understand the anti-allergy mechanisms regulated by RMFE, toluidine blue staining was performed on skin tissues (Figures 3A,B). Consequently, a statistically significant increase in activated mast cells was observed in the dermis of the VC group compared to the non-ovalbumin-treated area (CON: 7.3 ± 1.0, VC: 29.7 ± 6.5). However, these effects were reversed upon RMFE treatment (VC: 29.7 ± 6.5, T1: 7.3 ± 3.4). IgE overexpression and mast cell activation are hallmarks of cAD. A localized AD was induced in the dorsal region of canines using ovalbumin, and the expression of ovalbumin-specific IgE in the dorsal skin was measured (Figure 3C). The results revealed a statistically significant increase in ovalbumin-specific IgE expression in the atopic VC and T1 groups compared to the control (CON) site (CON: 5.8 ± 3.0, VC: 19.1 ± 2.5, T1: 18.3 ± 1.8). However, this increase was significantly reduced by RMFE treatment.

### 3.4 RMFE regulates Th2-related cytokine and inflammatory cytokine levels in canine AD

**Figure 4:**
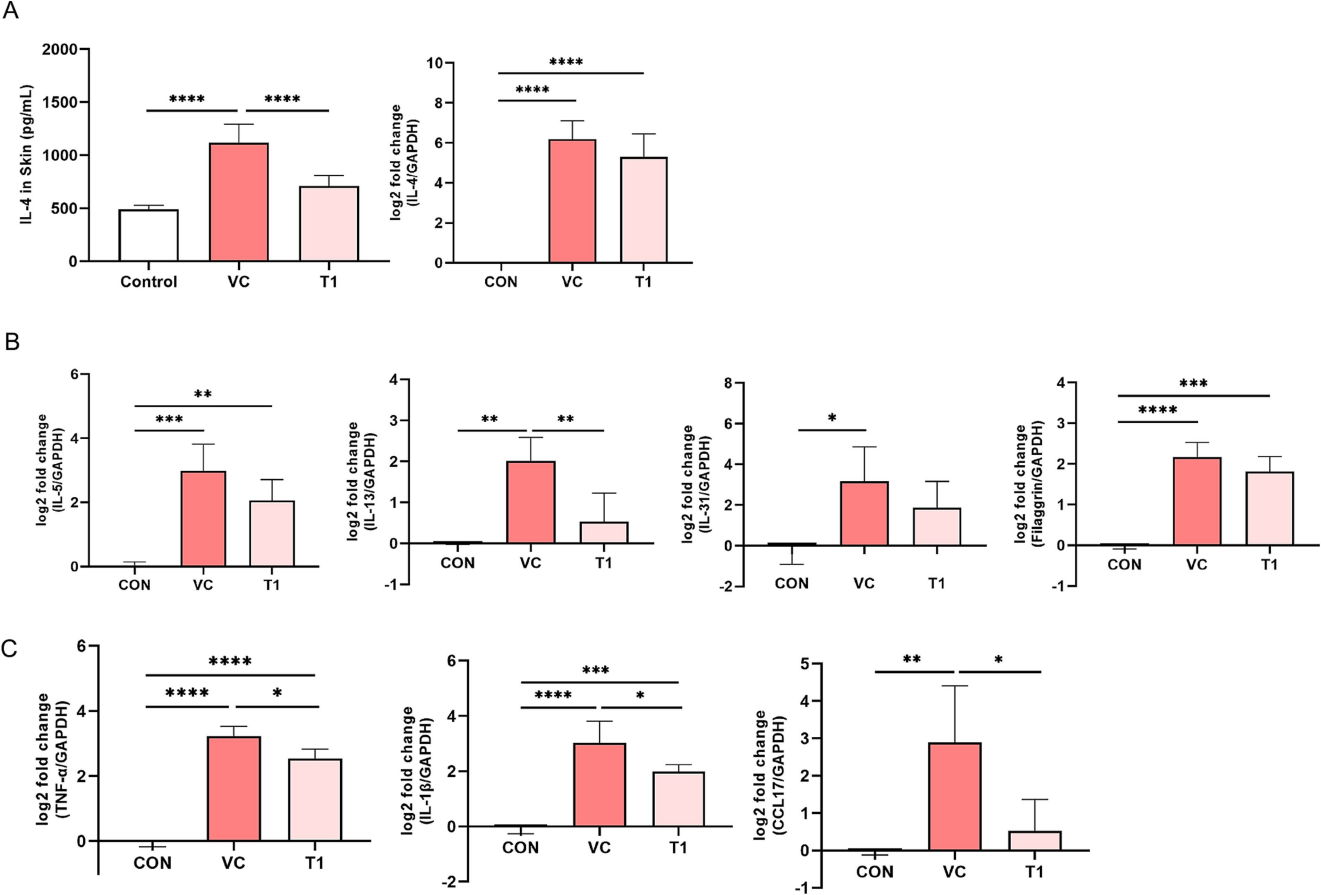
RMFE alleviates Th2-related cytokine and inflammatory cytokine levels in canine AD. Comparative analysis of Th2-related cytokine expression changes in canine AD after treatment in all groups. **(A)** IL-4 cytokine level expression (Left) and mRNA level expression (Right) analysis of untreated (CON) and ovalbumin-treated (VC, T1) skin. **(B)** Th2-related cytokines and skin barrier function-related cytokines mRNA expression analysis. **(C)** Inflammatory disease-related cytokines and chemokines mRNA expression analysis. *p*-values: * < 0.05, ** < 0.01, *** < 0.005, **** < 0.001; Bar graph: Mean ± SD.

To assess the effect of the oral administration of RMFE on Th2-related cytokines in the atopic dermatitis-induced dorsal region of the beagles, dorsal skin tissue was collected after the final oral dose and analyzed using ELISA and qRT-PCR. The dorsal skin tissue IL-4 ELISA results showed the following levels: CON (no ovalbumin): 489.9 ± 37.6 pg./mL; VC (ovalbumin): 1119.2 ± 172.0 pg./mL; and T1 (RMEF treatment): 709.4 ± 99.6 pg./mL. IL-4 expression was increased in all groups compared to the CON (without ovalbumin), with a statistically significant increase in cytokine levels in the VC (ovalbumin) group compared to the CON (no ovalbumin) group. However, these changes were reversed by RMFE treatment, resulting in a statistically significant decrease in IL-4 cytokine levels compared to the VC (ovalbumin) group. RT-qPCR analysis of IL-4 in the dorsal–ventral skin tissue showed an increase in all groups compared to the control group (CON (without ovalbumin): 0.0 ± 0.0, VC (ovalbumin): 6.2 ± 0.9, T1: 5.3 ± 1.1 log 2-fold) (Figure 4A). In addition, Th2-related cytokines (IL-5, IL-13, and IL-31) and filaggrin, which are important for skin barrier function, were significantly increased in the VC group compared with the control group. However, a statistically significant increase in IL-13 was observed upon RMFE treatment (Figure 4B). RT-qPCR analysis of pro-inflammatory cytokines (tumor necrosis factor-alpha [TNF-*α*] and IL-1β) and chemokine transcripts (TNF-α, IL-1β, and chemokine (C-C motif) ligand [CCL]17) in dorsal and ventral skin tissues showed a statistically significant increase in mRNA levels in all groups compared to the control group. However, this effect was reversed by a statistically significant decrease in the transcripts of TNF-α, IL-1β, and CCL17 in the RMFE group compared to the VC group, indicating an improvement in the inflammatory response in cAD (Figure 4C).

## 4 Discussion

RMFE has been implicated in AD; however, its effects on canines and the underlying mechanisms remain unclear. In this study, we administered RMFE orally for 2 weeks to beagles with ovalbumin-induced AD. RMFE improved clinical symptoms of cAD, modulated histopathological inflammation and immune cell infiltration, and altered Th2 effector cytokine levels. Furthermore, RMFE attenuated allergic responses in AD model dogs by reducing mast cell numbers, inhibiting their activation, and reducing IgE, especially ovalbumin-specific IgE production. RMFE modulates the Th2 immune response to AD and attenuates the AD-induced inflammatory response. Our findings suggest that RMFE is a potential therapeutic strategy to ameliorate atopy in companion animals.

The pathogenesis of AD remains complex. Allergen-specific effector T cells, which are biased toward predominantly Th2 cells, due to a Th1/Th2 imbalance, are a key mechanism in the pathogenesis of AD in both humans and dogs (24–26). Furthermore, cAD is associated with epidermal barrier dysfunction (27). Currently, animal models are the most utilized in the study of AD in both humans and pets (28–31). Due to the growing pet population, the development of disease models and therapeutic interventions for purpose-built animals is essential. Thus, we created a beagle AD animal model characterized by a Th2-dominant immune response to validate the safety of our previous study and to serve as a foundation for basic research purposes (22).

In this study, we induced AD by applying ovalbumin to the dorsum once daily for 2 weeks and repeated it twice in total. With the first ovalbumin application, erythema was observed at the treatment site in all animals by day 5. After 7 days, typical skin manifestations of cAD were observed, which included tanning and hyperpigmentation, scaling, and dryness. RMFE and vehicle (dextrin) were orally administered during the second ovalbumin application to assess the role of RMFE in atopy and its effectiveness in alleviating symptoms. Changes in body weight and food intake were observed to evaluate the stress caused by oral administration, which was not observed in all groups. Hematologic and blood biochemical changes were also observed. However, since we induced atopy locally in the dorsal region, no hematologic or blood biochemical changes associated with RMFE administration were observed.

Clinical observations revealed a decrease in skin lesions in the RMFE group compared to the control group, accompanied by a significant decrease in CADESI-04 scores. We also evaluated clinical symptoms of atopy using the CADESI-04. The animal model in this study did not reflect systemic atopy due to genetic predisposition. However, since we used ovalbumin to induce atopic dermatitis on the dorsal region, there was a mismatch with the scoring region criteria of CADESI-04, which are designed for atopic dermatitis caused by genetic predisposition. This was evaluated using the Draize dermal irritation scoring system in a previous study (22), but the clinical symptom scores did not match those seen in cAD. To address this, we used the modified CADESI-04 scoring system, which is used in dogs, to evaluate the right and left dorsal areas and then summed the results. In all animals, the CADESI-04 scores, which increased with the first dose of ovalbumin, were significantly reduced in the RMFE group after the last dose (Day 42) compared to the vehicle group. These findings suggest that RMFE can be assessed using CADESI-04 with simplified scoring sites and may help improve the cutaneous clinical manifestations of ovalbumin-induced canine-like AD.

The immune response in cAD is characterized by the activation and differentiation of CD4+ T cells (24, 32). CD4+ T cells, particularly Th2 cells, secrete cytokines such as IL-4, IL-5, and IL-13 in early cAD pathogenesis to induce an inflammatory response (33). Th2-dominant cytokines induce the expression of skin barrier proteins, leading to epidermal dysfunction (34). This accelerates the inflammatory response by promoting IgE production and the infiltration and activation of immune cells (35). In addition, the epidermal thickness is increased by various factors, such as mechanical stress due to AD and epidermal hyperplasia induced by increased pro-inflammatory cytokines, as confirmed by our histopathologic findings. RMFE inhibited the ovalbumin-induced increase in epidermal thickness and the expression of CD4+ T cells, suggesting a protective effect in cAD. We measured total IgE and ovalbumin-specific IgE in the skin of dorsal and ventral areas topically treated with ovalbumin to induce atopic dermatitis.

Mast cells are involved in the progression of cAD (36), with increased frequency and activation being hallmark symptoms (37, 38). The binding of allergen-specific IgE to mast cells via the FcεRI receptor primarily activates mast cells and accelerates allergic response (39). Subsequent re-exposure to the allergen induces degranulation of mast cells (40) and release of histamine (41, 42) and pro-inflammatory cytokines, leading to hypersensitivity and pruritus (43). In this study, we differentiated between total IgE and ovalbumin-specific IgE measurements. Total IgE, representing the overall IgE levels in the blood, was detected in a range of 0–300 μg/mL. In contrast, ovalbumin-specific IgE, which targets the specific allergen, was detected within a narrower range of 0–25 μg/mL levels in the blood, indicating its higher specificity in ovalbumin-induced cAD. Our established model demonstrated that ovalbumin-specific IgE was a more accurate biomarker in ovalbumin-induced cAD than total IgE. We hypothesized that RMFE alleviates cAD symptoms by inhibiting mast cell degranulation and infiltration. To test this hypothesis, we measured both total IgE and ovalbumin-specific IgE levels. Consistent with our hypothesis, we observed a statistically significant decrease in ovalbumin-specific IgE levels in the ovalbumin-sensitized model following RMFE treatment. This finding supports the potential mechanism of action for RMFE in reducing allergen-specific immune responses in cAD. These results suggest that ovalbumin-specific IgE may serve as a more precise indicator of allergen-specific responses in ovalbumin-induced cAD and that the therapeutic effect of RMFE may be mediated through the modulation of allergen-specific IgE production or mast cell activity.

Studies have shown increased mast cell density in the subepidermal and perifollicular compartments of animals with AD (44) and in various skin regions of AD dogs (45). Therefore, the increased mast cell frequency in the dorsal region of our animal model is not surprising. In this study, RMFE treatment inhibited the frequency of mast cells and IgE-mediated mast cell activation in the dermal layer of cAD dorsal skin lesions. Studies have shown that the number of mast cells increases with increasing cAD and type 2 dermatitis, which are associated with pruritus, the hallmark symptom of cAD. Mast cell activation by specific antigens induces the release of inflammatory mediators, recruiting inflammatory cells (e.g., T cells, neutrophils, and eosinophils) to the antigen-stimulated site due to the secretion of chemokines (CCL17, CXCL8, and CCL11). These cells, attracted by T cells, release pro-inflammatory cytokines, accelerate the skin inflammatory response, and exacerbate the Th2-dominant immune response (46–48).

RMFE inhibited the cytokine level of IL-4, which was elevated by ovalbumin in the skin, and downregulated the expression of Th2-related cytokines, such as IL-5, IL-13, and IL-31, shifting away from a Th2-dominant environment. Furthermore, these results suggest that RMFE has anti-allergic effects in cAD. We also observed that CCL17 mRNA levels, which were increased by ovalbumin, were reduced, and mRNA levels of TNF-*α* and IL-1β were downregulated by RMFE administration. The RMFE-induced inhibition of mast cell activation and frequency leads to suppression of the immune response in cAD, decreasing IgE levels and recruiting other inflammatory cells, such as eosinophils. Thus, the decrease in the release of inflammatory mediators suggests that RMFE has an anti-inflammatory effect in cAD.

In conclusion, our findings demonstrate that oral administration of RMFE effectively mitigates cAD by reducing the expression of CD4+ cells, mast cells, immune cells, and pro-inflammatory cytokines and chemokines. These results confirm RMFE as a potential therapeutic candidate for cAD treatment. However, to fully elucidate the mechanisms of RMFE in cAD treatment, further studies are needed. Specifically, future research should focus on assessing the effects of RMFE on regulatory cytokines and regulatory T cells (Tregs) and evaluating its broader therapeutic potential in the treatment of cAD.

## Data Availability

The original contributions presented in the study are included in the article/supplementary material, the raw data supporting the conclusions of this article will be made available by the authors, without undue reservation.
